# Adipose Tissue Inflammation

**DOI:** 10.3390/cells12111484

**Published:** 2023-05-26

**Authors:** Javier Gómez-Ambrosi

**Affiliations:** 1 Metabolic Research Laboratory, Clínica Universidad de Navarra, 31008 Pamplona, Spain; jagomez@unav.es; 2 Centro de Investigación Biomédica en Red-Fisiopatología de la Obesidad y Nutrición (CIBEROBN), Instituto de Salud Carlos III, 31008 Pamplona, Spain; 3 Obesity and Adipobiology Group, Instituto de Investigación Sanitaria de Navarra (IdiSNA), 31008 Pamplona, Spain

In recent decades, obesity has become one of the most common metabolic diseases. Excess adiposity increases the risk of developing type 2 diabetes (T2D), cardiometabolic diseases, dyslipidemia, fatty liver, and several types of cancer [1]. Much progress has been made in understanding the major regulatory pathways underlying adipose tissue inflammation, which represent one the main drivers of adipose tissue dysfunction and, consequently, of obesity-associated metabolic alterations [2].

This Special Issue presents recent advances in understanding the molecular processes that take place in adipose tissue inflammation; moreover, it discusses the impact of adipose tissue inflammation on systemic metabolic alterations associated with excess adiposity, as well as its repercussion in several pathological conditions.

Although obesity has traditionally been considered a single medical entity, in recent years, greater importance has been placed on phenotyping the different obesities in order to improve their clinical management [3,4]. In their cross-sectional and prospective study, Goldstein et al. analyze the usefulness of determining mast cell accumulation in human adipose tissue as a proxy of metabolic phenotyping [5]. They suggest that patients with obesity with high expression of mast cell genes exhibit a healthier metabolic phenotype than individuals with low expression. The authors also find that higher mast cell accumulation in adipose tissue in patients undergoing bariatric surgery is a predictor of greater weight loss after surgery. They conclude that a high number of mast cells defines a clinically favorable obesity phenotype [5].

Several studies discuss the molecular mechanisms that regulate the impact of adipose tissue inflammation. Lempesis et al. show that low physiological oxygen tension decreases the expression and secretion of proinflammatory adipokines in adipocytes obtained from patients with obesity, an effect that is not found in cells derived from donors of normal weight [6]. Kochumon and colleagues report that the adipose tissue expression of steroid receptor RNA activator 1 (SRA1) may represent a novel surrogate marker of metabolic inflammation through its association with Toll-like receptors (TLRs) [7].

Other studies provide interesting information regarding the regulation of inflammation in adipose tissue in mouse models. In one such study, Sandrini et al. study the effects of physical exercise on *BDNF* Val66Met mice, a model of increased adiposity associated with a proinflammatory and prothrombotic profile [8]. They find that four weeks of voluntary wheel running changes epididymal adipose tissue morphology and the expression of proinflammatory genes, inducing reversion of the prothrombotic phenotype; this suggests that a reduction in adipose tissue inflammation is important in promoting the positive effects of physical activity [8]. In another study, Mendes de Farias and colleagues find that daily melatonin supplementation for 10 weeks in mice on a high-fat diet reduces fat accumulation, adipocyte size, and the expression of proinflammatory adipokines in adipose tissue and the circulation; this suggests that melatonin could be considered as a therapeutic molecule for the treatment of obesity [9].

Adipokines and adipose tissue inflammation have been shown to play a role in several physiologic and pathophysiologic conditions [10,11], as stated in several studies included in this Special Issue. Morais et al. show that an adequate balance between adiponectin and leptin concentrations in human milk may regulate colostrum mononuclear cell activity, eliciting a more effective response against neonatal infection in breastfeeding infants [12]. Another adipokine, fatty acid-binding protein 4 (FABP4), implicated in the control of cellular lipid metabolism, is also involved in inflammation and the development of insulin resistance. In an exhaustive review, Trojnar et al. detail the different mechanisms by which FABP4 is involved in inflammation and insulin resistance and the potential role of this adipokine in T2D, gestational diabetes, and fatty liver, among other conditions [13]. Chang and Eibl describe the relevance of adipose tissue inflammation as an important driver of obesity-associated pancreatic ductal adenocarcinoma, and consider strategies aimed at reducing inflammation in this tissue as a weapon against this type of cancer [14]. In another interesting review, Cornide-Petronio and colleagues reinforce current knowledge regarding the interaction between the liver and adipose tissue during liver surgery. The scientific and clinical controversies in this area are reviewed, as are potential therapeutic approaches. The information provided could help to develop protective measures focused on manipulating the liver–visceral adipose tissue axis to enhance the postoperative results of hepatic surgery [15]. Finally, Demeulemeester et al. summarize scientific research on the link between obesity and COVID-19 severity, and analyze probable mechanisms that could help to understand why patients living with obesity exhibit an increased risk of serious consequences during COVID-19 [16].

In recent years, there have been significant advancements in the understanding of the cellular and molecular mechanisms involved in adipose tissue inflammation. Thanks to this progress, more tools and approaches are available for the treatment of obesity and T2D. However, in order to optimize the management of patients with obesity, more research needs to be conducted.
